# Changes in habitat selection patterns of the gray partridge *Perdix perdix* in relation to agricultural landscape dynamics over the past two decades

**DOI:** 10.1002/ece3.5114

**Published:** 2019-04-03

**Authors:** Clément Harmange, Vincent Bretagnolle, Mathieu Sarasa, Olivier Pays

**Affiliations:** ^1^ CNRS, UMR LETG, UFR Sciences Université d'Angers Angers Cedex 01 France; ^2^ Centre d'Etudes Biologiques de Chizé, UMR 7372, CNRS Université La Rochelle Beauvoir‐sur‐Niort France; ^3^ LTSER “Zone Atelier Plaine & Val de Sèvre” CNRS Beauvoir sur Niort France; ^4^ BEOPS Toulouse France; ^5^ Fédération Nationale des Chasseurs Issy‐les‐Moulineaux cedex France

**Keywords:** agricultural landscapes, farmland birds, gray partridge, habitat quality, habitat selection, habitat suitability maps, long‐term trends, *Perdix perdix*

## Abstract

1. Changes in agricultural practices have reshaped agricultural landscapes and triggered a drastic decline in spatial and temporal heterogeneity leading to changes in habitat quality and food resources for birds. However, the precise relationships between landscape changes, spatial and temporal heterogeneity, and habitat preferences in response to those changes remain poorly known.
2. We investigated patterns of habitat selection and causes for changes over the years 1997–2017 for the gray partridge *Perdix perdix*, an iconic farmland species which has experienced a severe decline since the 1950s. Using a long‐term (1997–2017) dataset collected over 435 km^2^, we modeled relationships between annual land‐cover maps and partridge sightings over 17 5‐year moving windows, assessing the effects of landscape metrics, the strength of the relationships, and the dynamics of habitat suitability.
3. We detected a shift in gray partridge habitat preferences over time, toward more risky habitats. Avoidance of predator reservoirs (woodlands and buildings) has weakened, and selection of human infrastructure, such as roads and tracks, has increased. Since 1997, the mean size of suitable patches has also decreased by about 26%.
4. We have interpreted these changes in habitat selection as being the result of decreasing habitat quality and the increasing prevalence of captive‐reared birds, currently released in their thousands in the study site.
5. *Synthesis and applications*. The gray partridge has not adjusted well to changes in farming practices, and the low, still decreasing, population density suggests that it is not sustainable, despite local initiatives and the investment in captive‐bird releases. We emphasize that efforts must be redirected toward habitat improvement to restore the density of refuge cover, insects, and seeds in the landscape, hunting management to ensure self‐sustainable populations and massive releases of high‐quality birds. Only integrated local management, involving hunters, farmers, gamekeepers, and scientists can ensure the recovery of this species.

## 1 INTRODUCTION

For decades, evidence has accumulated that the main driver of the decline in farmland biodiversity lies in the rapid postwar changes in agricultural practices (Benton, Vickery, & Wilson, 2003; Krebs, Wilson, Bradbury, & Siriwardena, 1999; Robinson & Sutherland, 2002). Agricultural intensification and specialization have reshaped and simplified agricultural landscapes (Benton et al., 2003). The resulting loss of spatial and temporal heterogeneity has degraded habitat quality and food resources, resulting in severe declines in European farmland birds (Benton et al., 2003; Chamberlain, Fuller, Bunce, Duckworth, & Shrubb, 2000; Robinson & Sutherland, 2002). Though agricultural landscapes cover about 40% of the earth's terrestrial biomes, and are highly dynamic, long‐term changes in agricultural landscapes have seldom been quantified: only seven (3%) in a review of 244 studies assessing land‐cover changes (Uuemaa, Mander, & Marja, 2013). The scarcity in such studies comes from the challenge of accessing land‐cover data over the long term (Bertrand, Burel, & Baudry, 2016) as well as the complexity of analyzing interacting landscape features, each with their own spatio‐temporal dynamics. For instance, in intensive agricultural landscapes, buildings, roads, hedgerows, and woodlands have broadly remained stable over the years, while crops change annually in a quasi‐stochastic spatial pattern of crop rotation (Bretagnolle et al., 2018b). As a result, very few studies have investigated the temporal changes in habitat selection by farmland birds associated with the temporal changes in the agricultural landscape (but see Brambilla et al., 2010), despite the behavioral process of habitat selection being critical in determining population dynamics, survival, and productivity (Jones, 2001).

The gray partridge *Perdix perdix* L. used to be one of the most common farmland birds in Europe, but has been in a steep, widespread decline since the mid‐20th century (Aebischer & Potts, 1994; Gée, Sarasa, & Pays, 2018; Sotherton, Aebischer, & Ewald, 2014). In many European countries, numbers are at <10% of their prewar level (Aebischer & Kavanagh, 1997). For example, the drop in abundance was estimated at 92% between 1970 and 2015 in the UK, and it is now a red‐listed species in the UK Birds of Conservation Concern (Hayhow et al., 2017). Despite a decline of 23% in abundance from 1989 to 2015 (Vigie‐Nature, 2018) and 20% in the distribution range from 1985 to 2013 (Comolet‐Tirman et al., 2015), gray partridge is still considered as “Least Concern” in France. The decline was attributed to three main causes, all linked to the decrease in habitat quality resulting from agricultural intensification: the loss of breeding habitat, the decrease in availability of insects for chicks, and the concentration of partridges and predation pressure in remaining habitats (Aebischer & Ewald, 2012). Gray partridge habitat selection has been well studied: At a large scale, the species avoids woodlands, associated with high predation risk, as woodlands are predator reservoirs (Dudzinski, 1992), and also avoids the proximity of buildings with their predation risks from cats and mustelids (Reitz, Le Goff, & Fuzeau, 2002). At a more local scale, during the breeding season, gray partridges were found to be attracted by cereal cover, with a high nesting success rate, (Bro et al., 2013; Bro, Reitz, & Mayot, 1998), rape and grassy covers (Birkan, Serre, Skibnienski, & Pelard, 1992; Bro et al., 2013), crop diversity (Reitz et al., 2002), and linear features such as hedgerow fragments, roads, and tracks (Blank, Southwood, & Cross, 1967; Bro et al., 2013; Potts & Aebischer, 1991), even though these linear features are associated with lower nesting success (Bro et al., 1998). However, with a few exceptions (Reitz et al., 2002; Ronnenberg, Strauß, & Siebert, 2016), in most studies, the effects of landscape features have been studied independently from each other so that the relative contributions of different landscape features on gray partridge habitat selection remain unknown. Even less is known about potential changes in patterns of habitat selection over time in highly dynamic agricultural landscapes.

**Figure 1:**
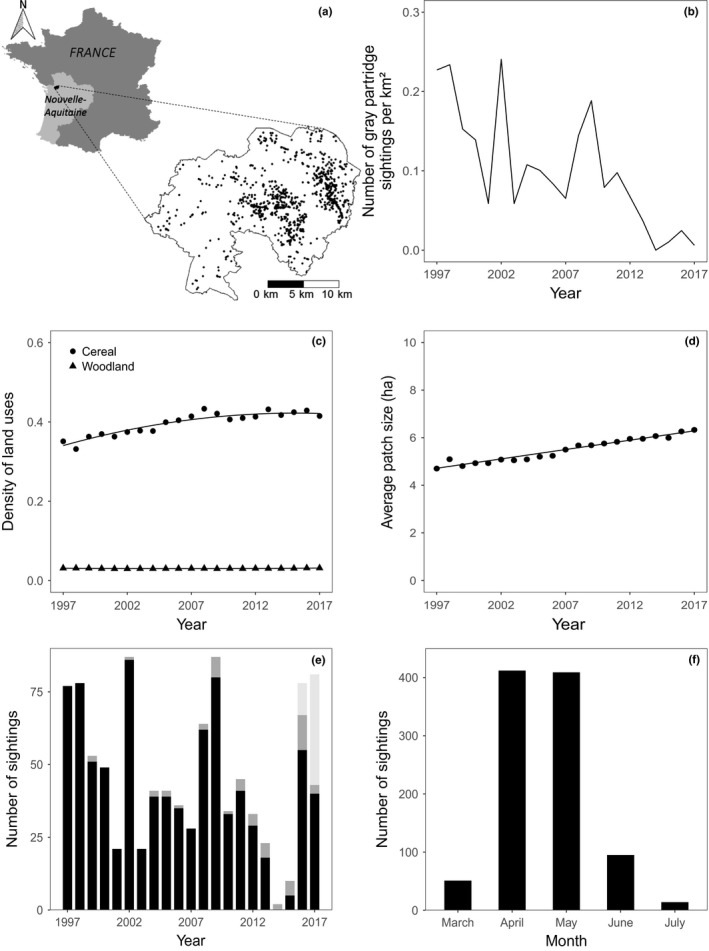
(a) Location of the Long‐Term Socio‐Ecological Research (LTSER) platform “Zone Atelier Plaine & Val de Sèvre” in France. Black dots inside the LTSER represent gray partridge sightings recorded from 1997 to 2017. (b) Density of gray partridge opportunistic sightings (see Methods) from 1997 to 2017. (c) Density of cereals (time varying) and woodland (stable) from 1997 to 2017. Solid lines represent significant trends from generalized least squares (GLS) model with autoregressive moving average (see Table S1 for statistical details). (d) Average size of crop patches from 1997 to 2017 on the LTSER. Solid lines represent significant trends from GLS models with autoregressive moving average. (e) Gray partridge sightings each year between 1997 and 2017 for each dataset (black: opportunistic sightings; dark gray: point counts; light gray: point counts with call playback). (f) Distribution of gray partridge sightings recorded in each month of the year from 1997 to 2017

Here, we investigated long‐term trends in gray partridge habitat preferences in a French farmland landscape, through a multistep approach using data collected annually in an area of 435 km^2^ over 21 years (from 1997 to 2017). We first identified the key landscape features that explain the gray partridge occurrence over 1997–2017 studying the variable contributions to the probability of occurrence. Contributions reflect the importance of variables to explain (or predict) the occurrence of a species and are commonly used to identify the key drivers of species distribution (e.g., Bellard, Leroy, Thuiller, Rysman, & Courchamp, 2016; Wilson, Sexton, Jobe, & Haddad, 2013). Secondly, to explore whether and how the habitat selection patterns have varied with changes in the landscape, we quantified changes in the relative contributions and effect of landscape features on gray partridge occurrence with time, over 17 5‐year moving windows. Thirdly, we investigated whether overall habitat suitability for gray partridge has changed over time, using as reference the oldest habitat preferences of gray partridge available (i.e., models based on data from 1997 to 2001; see Pearman, Guisan, Broennimann, & Randin, 2008). In this study area, significant changes in land‐cover, for example, a 20% increase in cereal cover since 1994 (Bretagnolle et al., 2018b) and a decrease in insect availability (Bretagnolle et al., 2011) have been recorded while observed partridge densities have strongly decreased (Figure 1b) despite huge annual releases of captive‐reared birds to maintain local partridge populations (Bro & Crosnier, 2012). Given this observed decline in the study population associated with the relatively stable amount of captive‐reared gray partridges annually released in France from 1995 to 2015 (around 2 million individuals in Bro, 2016), we therefore, assumed that the population in our study area has changed from mainly wild birds in the nineties to mainly released captive‐reared birds currently. We thus suggested the massive releases of captive‐reared birds as a potential driver of changes in the pattern of habitat preferences. Indeed, we might expect that naïve partridges would be less likely to avoid predator‐rich habitats. If there has been a general decline in habitat quality, partridges may select the least altered or highest quality habitats. Although habitat selection is density dependent (Morris, 1989), over the same period the population of partridges has strongly declined, so that habitat preferences may have remained targeted to the highest quality habitats.

## 2 MATERIAL AND METHODS

### 2.1 Study site and environmental layers

The Long‐Term Socio‐Ecological Research platform (LTSER) “Zone Atelier Plaine & Val de Sèvre” is located in Central Western France, Nouvelle‐Aquitaine region (Figure 1a, Bretagnolle et al., 2018b). This area is characterized by an intensive agricultural landscape for cereal production. Land‐cover has been exhaustively surveyed annually from 1994 onward using more than 40 crop categories. Winter cereals largely dominate (41.5%, mean value calculated over the years 2009–2016), followed by sunflower (10.4%), maize (9.6%), rape (8.3%), and meadows (13.5%). Woodland fragments represent 2.9% of the study area (excluding the Chizé forest) and 9.8% of built‐up areas (including isolated buildings). A preliminary analysis of landscape features showed that the density of hedgerows, buildings, and woodlands has varied little, whereas the areas of cereals, meadows, and rape, as well as crop diversity and the size of crop patches, have changed far more over the period (Figure 1c,d, Supporting Information Figure S1 and Table S1 for statistical details). In addition, crop locations change annually because of crop rotation.

The vector based GIS was first transformed into a grid of 200 × 200 m^2^ cells (4 ha), using R (R Core Team, 2017) and QGIS (Quantum GIS Development Team, 2017), excluding all pixels that had less than half their area intersecting with the study area. The pixel size was chosen to have a meaningful fine scale resolution in relation to landscape dynamics that was close to the average field size (Bretagnolle et al., 2018b). Landscape features already identified in the literature as being selected by gray partridges were chosen for analyses as potential predictors of the gray partridge occurrence: cereals, rape, meadows, hedgerows, roads, and tracks (Birkan et al., 1992; Blank et al., 1967; Bro et al., 2013), and woodland and buildings, features that partridges avoid (Dudzinski, 1992; Reitz et al., 2002). The landscape metrics (i.e., layers assessing landscape features) used in the analyses comprised the densities of various landscape features (the area in ha, or length in meters, of each landscape feature divided by area of the pixel in ha), the crop diversity (Shannon‐Wiener diversity index calculated for five main crops (cereals, rape, meadow, maize, and sunflower), and “other crops”), the mean size of the crop patches (mean area of the patches of the main crops intersecting each pixel), and the distances from features avoided by partridges (distances from the center of the pixel to the closest woodland and closest building; Supporting Information Table S2). Each of these seven metrics was calculated for each of the 21 years from 1997 to 2017 giving 147 map layers. Roads, tracks, and hedgerows have remained fairly stable over the period (Supporting Information Appendix S1, Figure S1) so one map layer was used for roads and tracks and three layers for hedgerows in 2006, 2011, and 2014 when aerial photographs were available. A total of 151 layers were, therefore, included.

### 2.2 Partridge data

In total, there were 989 sightings of gray partridges in three distinct datasets covering 1997 to 2017 (Figure 1e). The largest dataset (90% of total data) comprised opportunistic sightings either when ornithologists working in the LTSER were studying other bird species (Bretagnolle et al., 2018b) and saw partridges, or during the land‐cover surveys carried out twice a year systematically over the entire study site. Each year, opportunistic sightings were reported daily between late March and late July (see Figure 1f). In this dataset, the observation effort is not standardized and reporting is not systematic. In the second dataset, with 5% of total sightings, observation pressure is standardized as this dataset collates the results from systematic bird point counts since 1995 (see Brodier, Augiron, Cornulier, & Bretagnolle, 2014 for methods). The number of point counts has increased from 160 (1995–2008) to about 450 since 2009, spread over the whole of the LTSER (Bretagnolle et al., 2018a). Count duration was either 5 or 10 min (see Brodier et al., 2014) between 7:00 a.m. and 11:00 a.m., without call playback. The third dataset, with 5% of total sightings, collates the results of standardized call playback counts specifically for both gray partridges and red‐legged partridges *Alectoris rufa* L., in March–April 2016 (140 sites) and 2017 (275 sites), either in the morning (from 7:00 a.m. to 11:00 a.m.) or evening (from 5:00 p.m. to 9:00 p.m.). Count duration was 10 min. In all three datasets, the location of the birds, the date and time, and the number of birds were recorded. We restricted our analyses to data collected during the breeding season, from late March until July (Figure 1f), using the data in all three datasets.

### 2.3 Species distribution models

A multistep approach using species distribution modeling was carried out to (a) identify key landscape features and their effect on the gray partridge occurrence over the entire period 1997–2017; (b) explore trends in the contribution of key features and in their effect over 17 successive 5‐year moving periods; (c) assess changes in habitat suitability for gray partridge over time.

#### 2.3.1 Modeling process

The following modeling process was first applied over the entire study period (1997–2017) to investigate the general pattern of habitat selection. Each landscape metric was pooled over the entire study period 1997–2017 (calculating an average value by pixel). To perform the trend analysis assessing changes in habitat selection patterns, the modeling process was also applied using a 5‐year moving window, giving 17 successive 5‐year periods from 1997–2001 to 2013–2017, to have between 100 and 300 sightings at each time step (see Barbet‐Massin, Jiguet, Albert, & Thuiller, 2012). Each landscape metric was thus also pooled by 5‐year periods.

We began by selecting uncorrelated landscape metrics to ensure the validity of the predictions (Barbet‐Massin & Jetz, 2014) using the method described by Leroy et al. (2013), resulting in nine uncorrelated explanatory variables for the dataset with 5‐year moving windows (Supporting Information Table S2); however, the rape density was excluded for modeling the dataset aggregated over the whole period (Supporting Information Appendix S2). Secondly, to select the modeling technique (Elith, Ferrier, Huettmann, & Leathwick, 2005), we used version 2.0 BIOMOD multimodel platform (Thuiller, Lafourcade, Engler, & Araújo, 2009) implemented in R. This can (a) use heterogeneous data from different counting methods (Farashi & Shariati, 2017; Jackson, Gergel, & Martin, 2015) and (b) compare the most frequently used modeling techniques (Barbet‐Massin et al., 2012; Monnet, Hingrat, & Jiguet, 2015; see Supporting Information Appendix S3). As these modeling techniques require presence and absence data but as our dataset did not contain true absences (the absence of a sighting does not imply the absence of partridges), we generated 100 replicates of 1,000 random pseudo‐absences to obtain reliable confidence intervals (Barbet‐Massin et al., 2012; Supporting Information Appendix S3 gives the methods and numbers of pseudo‐absences). Generalized linear models (GLM, binomial response variable, logit link) were selected for the rest of the analyses as they showed a high true skill statistic (TSS = sensitivity + specificity−1; Allouche, Tsoar, & Kadmon, 2006) and high sensitivity (Supporting Information Appendix S3). For each period, the models were calibrated on a random subset of 70% of the presence/pseudo‐absence data and then cross‐validated using the remaining 30%: This cross‐validation was performed three times (Thuiller, Lafourcade, & Araujo, 2009). The TSS was used to evaluate the predictive performance: TSS ranges from −1 to 1, where 1 represents perfect agreement while scores ≤0 represent a performance no better than random (Allouche et al., 2006). For each period, the mean TSS was calculated averaging each model's TSS. The final calibration of each model used 100% of the available data.

#### 2.3.2 Assessing the contribution of each landscape metric

The average contribution of each landscape metric on the probability of the occurrence of gray partridge was determined for the entire period 1997–2017, and for each of the successive 5‐year periods using the *biomod2 variables importance* function. First, a standard prediction of gray partridge probability of occurrence is made across all pixels of the study area from a model calibrated with all explanatory variables. Then, the values of a given variable are randomly permutated across pixels and a new prediction is made. The contribution of the given variable to the prediction of the probability of occurrence is 1 less the correlation between the standard and the random predictions: the higher the correlation, the weaker the contribution or predictive power of the variable to explain the species occurrence (Leroy et al., 2013). Ten randomizations were used for each variable with a threshold of 0.1 to distinguish between strong and weak contributions (Leroy et al., 2013) which corresponded to the mean importance for all variables (Capinha & Anastácio, 2010).

#### 2.3.3 Effect of landscape metrics on the gray partridge occurrence

The relationship between landscape metrics and the probability of the occurrence of gray partridge was assessed using the average response curves from GLMs calibrated on 100% of the data. The 95% confidence intervals were calculated as a measure of the uncertainty for 100 GLM runs. The range of habitat values selected by partridges was drawn based on the average cutoff calculated for each period (W. Thuiller, pers. comm., 2016). The cutoff is the threshold maximizing TSS and is calculated for each model run under *biomod2* (Leroy et al., 2013; W. Thuiller, pers. comm., 2016).

#### 2.3.4 Trends in the relation between partridge occurrence and landscape metrics

To test whether time (explanatory variable) had a significant effect on the contribution (response variable) of landscape metrics, we used a generalized least squares (GLS) model using the *nlme *package. To handle autocorrelation in our time series, we run an autocorrelation function on each dependent variable to identify the time lag after which the non‐autocorrelation assumption was confirmed at 95% confidence level (Shumway & Stoffer, 2011). Then, GLSs were implemented with an autoregressive moving average (ARMA) term in which the moving average (MA) errors were accounted for using the maximum time lag determined in the previous step. The lack of sequential autocorrelation in the residuals was checked using the *acf* function. The 5‐year moving average windows allow a smoothing effect in the shape of the relationship between time and variable contribution. Finally, we included a quadratic time term in the GLS models and used backward stepwise selection procedure (Faraway, 2005).

The curves for each of four contiguous windows (1997–2001, 2002–2006, 2007–2011, and 2012–2016) were also drawn for each landscape metric that both made a large contribution and changed significantly with time.

#### 2.3.5 Habitat suitability maps and trends in habitat quality

We forecasted habitat suitability maps for the periods 1998–2002 to 2013–2017 from the models fitted to the period 1997–2001 to assess how suitable habitats formerly selected by partridges have shifted over time. Habitat suitability was mapped as the probability of occurrence from an ensemble model, averaging all GLMs with TSS > 0.4 (Engler et al., 2011; Zhang et al., 2015), each one weighted by its TSS. Then, we calculated Schoener's D (Schoener, 1968 reviewed by Warren, Glor, Turelli, & Funk, 2008) as a measure of similarity in the geographical space between the habitat suitability maps of each period and the earliest one (1997–2001).

$$D\left(p_{X},p_{Y}\right)=1-\frac{1}{2}\sum_{i}\left|p_{X,i}-p_{Y,i}\right|$$

where *p_X_*_,_ *_i_* and *p_y_*_,_ *_i_* represent the probabilities of occurrence of the gray partridge in the pixel *i* for the periods *X* and *Y*. The maps were then transformed into binary maps of suitable versus unsuitable areas, the threshold being the cutoff of the models calibrated on data of 1997–2001 (Leroy et al., 2013). The averaged value of habitat suitability index (i.e., the probability of occurrence) and the mean size of suitable habitat patches were calculated for each period.

## 3 RESULTS

**Figure 2:**
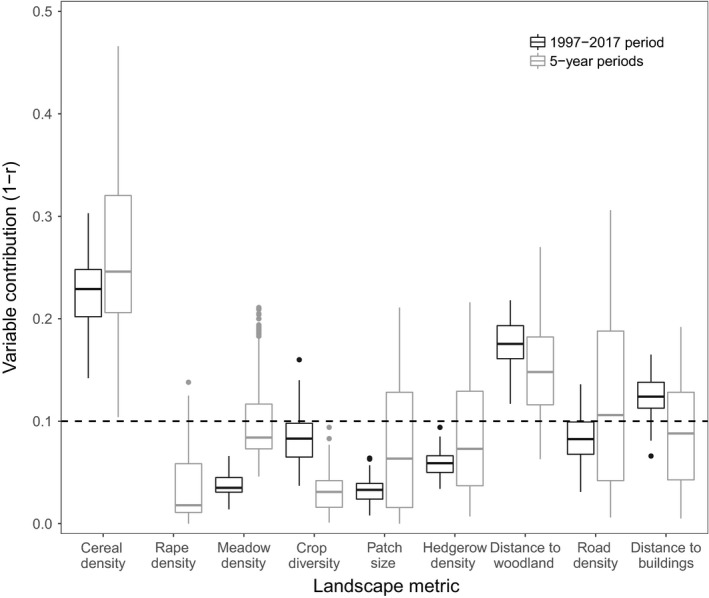
Contributions of landscape metrics to models calibrated on the whole 21‐year period (black) and cumulative contributions to models calibrated on the four contiguous 5‐year periods (1997–2001, 2002–2006, 2007–2011, 2012–2016). Rape density was discarded from the analysis for the whole 21‐year period as it was correlated with cereal density. The dashed line represents the threshold used to distinguish the landscape metrics that contributed most to the model (Capinha & Anastácio, 2010; Leroy et al., 2013)

**Figure 3:**
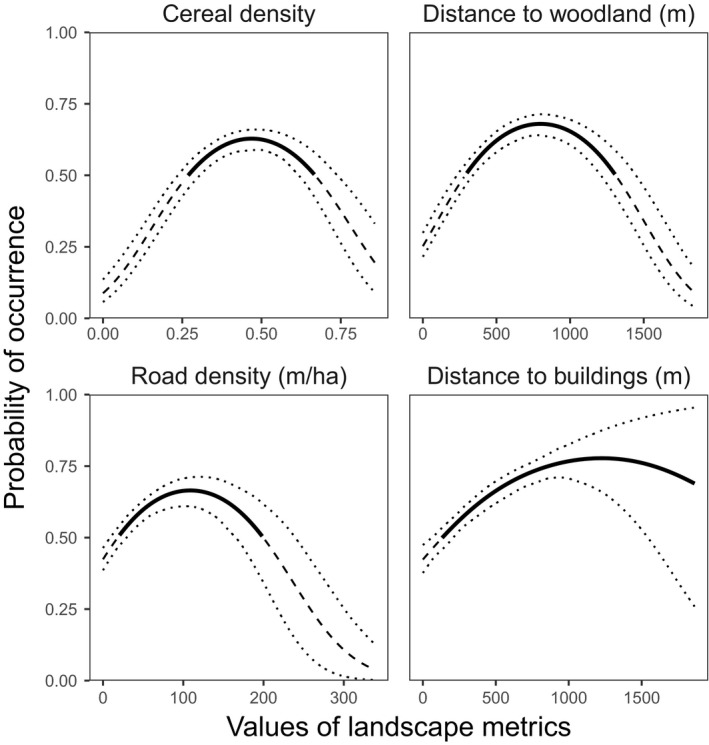
Probability of occurrence of gray partridge as a function of the landscape metrics that contributed most. Dashed lines represent the mean probability of occurrence below the cutoff, dotted lines represent 95% confidence intervals, and solid lines represent the range of values selected by gray partridge (above the cutoff, see Methods)

First, we investigated the key landscape metrics explaining the gray partridge occurrence over the study period (1997–2017), and their effect on gray partridge occurrence. Of the nine candidate explanatory variables, cereal density, distance to woodlands, and distance to the nearest building were the metrics with the highest contributions when fitting to the aggregated dataset for 1997–2017 (contributions >0.1, Figure 2; Supporting Information Table S3). Most response curves were quadratic: Moderate values of cereal density (25%–65%) and distance to woodlands (300–1,310 m) were preferred by partridges while extreme values were avoided (Figure 3). Partridges also preferred being more than 125 m from a building (Figure 3).

For the contiguous 5‐year periods, most of the metrics had similar contributions to those for the whole dataset (Figure 2; compare black and gray boxes), though the contribution of the distance to buildings was below the 0.1 threshold and the contribution of the road density increased above the 0.1 threshold (Figure 2). The response curve for road density was also quadratic with a preference for densities between 20 m/ha and 200 m/ha (high road densities were mainly in built‐up areas; Figure 3).

**Figure 4:**
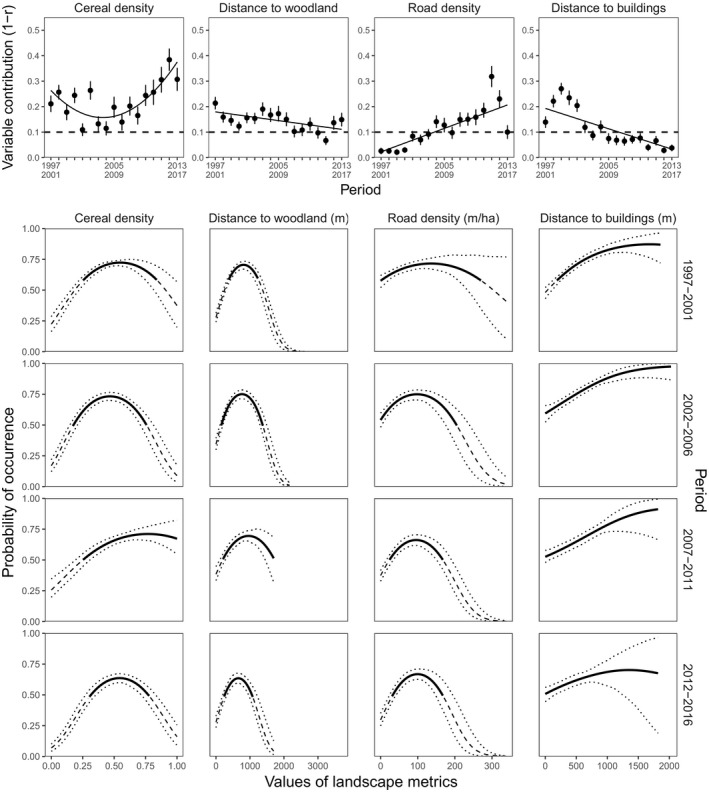
Top: contributions (black dots) of landscape metrics in the 5‐year moving window. Solid lines show the trends from the GLS model with autoregressive moving average (see Table S4 for statistical details). The dashed line represents the threshold used to distinguish the landscape metrics that contributed most to the models (Capinha & Anastácio, 2010; Leroy et al., 2013). Error bars correspond to the standard deviation of the contributions. Bottom: Probability of occurrence of gray partridge as a function of the landscape metrics for the four contiguous 5‐year periods (1997–2001, 2002–2006, 2007–2011, 2012–2016). Dashed lines represent the mean probability of occurrence below the cutoff, dotted lines represent 95% confidence intervals, and solid lines represent the range of values selected by gray partridge (above the cutoff, see Methods)

Then, we performed a trend analysis to investigate whether the importance of the key landscape metrics and their effect on gray partridge occurrence have changed with time. The trend analysis of the 17 5‐year moving windows showed that there were significant variations over time in the contributions of some metrics (Supporting Information Table S4). For example, the contribution of cereal density was variable in the first few years and then increased in the last 15 years. This increase in contribution was associated with a decrease of sightings in areas with low cereal density unselected by partridges (13% of sightings in area with cereal density <0.25 in 1997–2001; 7% in 2012–2016; Supporting Information Table S5). The contribution of road density increased over the whole period with the selected range contracting from 0–270 m/ha in 1997–2001 to 35–165 m/ha in 2012–2016 (Figure 4, Supporting Information Table S6). Recently, partridges appeared to avoid woodlands and to buildings less, as the contributions of these metrics decreased (Figure 4, Supporting Information Table S4). Currently, partridges prefer areas closer to woodlands (distance to woodlands is 19.5% shorter) than at the start of the study period (820 m in 1997–2001 decreasing to 660 m in 2012–2016, Figure 4, Supporting Information Table S6). This result is also consistent with raw numbers of partridge sightings (15% of sightings at a distance to woodlands <250 m in 1997–2001; 22% in 2012–2016; Supporting Information Table S5). Partridges were less likely to avoid buildings in recent years than in 1997–2001 when they avoided areas closer than 160 m to a building (Figure 4, Supporting Information Table S6). In 1997–2001, 13% of sightings were collected at a distance lower than 160 m to buildings, against 21% in 2012–2016 (Supporting Information Table S5). The contribution of the size of crop patches decreased while that of hedgerow density increased, but both contributions were small (Supporting Information Figure S2, Table S4).

**Figure 5:**
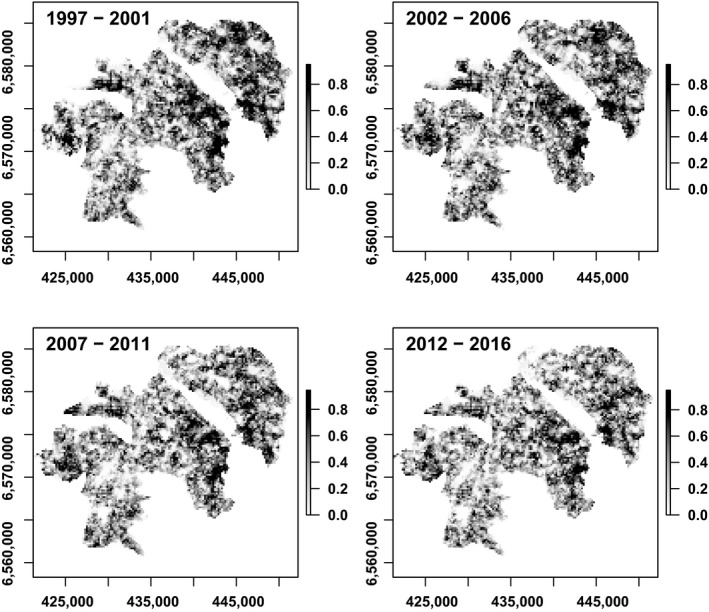
Predicted habitat suitability index of the LTSER for the four 5‐year contiguous periods. *X* and *Y* axes are the coordinates (Lambert 93, EPSG:2154)

Finally, we assessed the trends in habitat suitability with time. Habitat suitability maps calibrated on the first period (1997–2001) and forecast using the 1998–2002 to 2013–2017 metrics, suggested a general decrease by about 26% in the mean size (from 26.4 ha in 1997–2001 to 19.6 ha in 2013–2017) and a slight decrease in the mean suitability index (from 0.696 in 1997–2001 to 0.682 in 2013–2017) of suitable patches for partridges (Figure 5). Schoener's D statistic assessing the similarity of the habitat suitability maps between each 5‐year period and the first one decreased by 18.5% over time (Figure 5, Supporting Information Table S7). Even though the quality of our models remained relatively low (TSS = 0.42 ± 0.03 for the seventeen 5‐year periods, Supporting Information Table S3), it was high enough to be considered useful being above the recognized threshold of 0.4 (Engler et al., 2011; Zhang et al., 2015).

## 4 DISCUSSION

Despite many releases of captive‐reared birds in the study area, the population of gray partridges has suffered a drastic decline over the last 21 years. While we observed a general decrease in the size and suitability index of suitable patches, our study showed that occurrence of partridges has changed with landscape features.

Considering the overall 21‐year period (1997–2017), the density of cereals, the distance to woodlands, and the distance to the nearest building were identified as the main contributors or key drivers of gray partridge occurrence (Bellard et al., 2016; Wilson et al., 2013). Cereals is well known as the main breeding cover selected by gray partridges (69% of nests in Bro et al., 2013), associated with high nesting success (Bro et al., 1998). However, the response curve shows saturation, indicating a need for complementary habitats, for example, rape for food (Birkan et al., 1992). High contributions of distances to woodlands and buildings were expected because of avoidance of carnivore reservoirs (Dudzinski, 1992; Reitz et al., 2002; Reitz & Mayot, 1999), and woodlands have already been identified as the main driver of gray partridge distribution at local district scale (Ronnenberg et al., 2016). For the four 5‐year contiguous periods, the distance to buildings was less important while road density was more important. Partridges avoided low road densities, as nesting sites are frequently close to roads (25% of nests located in cereals, Bro et al., 2013), but they also avoided high road densities, either because of predation risk (Reitz & Mayot, 1999) or disturbance by traffic (Fahrig & Rytwinski, 2009). Although the population was mainly captive bred, this suggests that the partridges showed patterns of habitat selection that were driven by well‐known ecological requirements.

An unexpected result is that there was a strong increase in the contribution of cereals to explain the gray partridge occurrence over the past 15 years. As the cereal density has increased by about 20% of the preexisting density observed in 1997 (Bretagnolle et al., 2018b), this cover has become more widespread, and a subsequent decrease in the contribution of cereals might be expected. The selection and high nesting success of cereals (Bro et al., 2013, 1998) associated with the depletion of gray partridge densities may explain this result. The depletion would have been higher in the low and unselected densities, as supported by the decreasing number of sightings in areas providing low cereal densities. The other main result is the decreasing contributions of distances to woodlands, buildings, on the partridge occurrence, associated with increasing proportions of partridge sightings close to these predator‐rich features. While these landscape features have remained fairly stable over time, this suggest that partridges became less likely to avoid woodlands and buildings (predator reservoirs), and roads (high exposure to predation; Reitz & Mayot, 1999), though they were expected to select the best quality habitats. Nonetheless, lower avoidance of roads could also be partly explained by the decreasing partridge density associated with the opportunistic nature of sightings, usually collected from roads. Such shifts in gray partridge habitat preferences toward more risky habitats associated with lower survival expectancy have already been described and explained by captive rearing of partridge and other galliformes, lacking antipredator behavior (Rantanen, Buner, Riordan, Sotherton, & Macdonald, 2010a,2010b; Sokos, Birtsas, & Tsachalidis, 2008). Furthermore, captive‐reared birds could also suffer from natal habitat preference induction (Stamps & Swaisgood, 2007), encouraging them to select anthropogenic features similar to those where they were reared. However, as habitat selection impairing fitness has also been documented in wild gray partridges (Bro, Mayot, Corda, & Reitz, 2004) and other farmland birds (e.g., the yellow wagtail *Motacilla flava* in Gilroy, Anderson, Vickery, Grice, & Sutherland, 2011), there may be another explanation. This shift in habitat selection pattern could be linked to a behavioral adjustment to the decreasing habitat quality and food availability. For instance, over the last 30 years, seed availability has dropped faster in field cores than at field edges (Fried, Petit, Dessaint, & Reboud, 2009). Partridges could therefore avoid roads less, as roadside habitats may provide more insect and seed food (Hopwood, 2008; von der Lippe, Bullock, Kowarik, Knopp, & Wichmann, 2013), and higher seed abundance has already been linked to higher densities of farmland birds, including gray partridge (Moorcroft, Whittingham, Bradbury, & Wilson, 2002). This hypothesis could also explain the increase in the contribution of cereal density as the decrease in landscape diversity would encourage partridges to prefer this cover which is associated with a high nesting success rate (Bro et al., 2013,1998). The continuous drop in habitat quality caused by agricultural intensification could have increased the concentration of prey and the predation pressure on the remaining suitable patches (Aebischer & Ewald, 2012). This raises concern about low‐density populations, which may be particularly damaged as a result of a behaviorally mediated Allee effect: There is no competition for space and individuals are free to select their preferred, though possibly associated with lower survival, habitats (Kokko & Sutherland, 2001).

### 4.1 Implications for management

The gray partridge study population, with a current density of around 1 male (or pair) per 400 ha, may not be sustainable. Additionally, the observed changes in habitat selection toward more risky habitats could impair the situation and may be exacerbated by releases of captive‐reared birds, documented to suffer deficiencies in antipredator behavior, poor survival, and breeding success (Parish & Sotherton, 2007; Rantanen, Buner, Riordan, Sotherton, & Macdonald, 2010b). Therefore, initiatives releasing captive‐reared birds (about 250 birds per 1700 ha, the mean area of a local community) may actually precipitate, rather than mitigate, the fate of local populations, since these naïve birds outnumber wild individuals (Reitz, 2003; Sokos et al., 2008). Furthermore, these costly release programs, about €55k to €60k each year in the LTSER (36 hunting associations × 250 birds × €6.5), have so far failed to help the population recover, as numbers are still declining in the LTSER and the surrounding countryside. Extrapolating to other gamebird species (€55–€60k per year for red‐legged partridges and €45–€50k for ring‐necked pheasants), the costs of supplementing the local populations of gamebirds can be estimated at €155–€170k per year in our study area, with no perceivable improvement in population sustainability.

This does not imply that reinforcement is an invalid strategy, since several conservation programs would have sustainably increased partridge densities (Bro, 2016; Browne, Buner, & Aebischer, 2009; Buner & Aebischer, 2008). These programs, however, included supplementary feeding, reestablishment of refuge covers and insect‐rich habitats for chicks (set‐asides, grassy strips along field edges, field divisions, game covers), and nest protection or predation control when needed to accelerate population recovery (Aebischer, 2009; Aebischer & Ewald, 2012; Bro, 2016). In our study area, money saved by reducing by half the amount of captive‐reared bird released (i.e., by €80k) might be reallocated to create, for example, 270 ha of set‐asides or wildlife covers, or a maintenance of 40 km^2^ (i.e., 1/5 of the cereal area) of stubble fields until the end of the chick‐rearing period. In addition, the origin of the released birds was identified as a critical factor in success whether the aim was game management or conservation (Fischer & Lindenmayer, 2000; Griffith, Scott, Carpenter, & Reed, 1989). For instance, Sokos et al. (2008) reported a failure to re‐establish a pheasant population in a release program involving 3,000 captive‐reared birds, while the release of 1,000 wild‐caught individuals succeeded.

In the case of the gray partridge, we believe that we need to change from releasing captive‐reared gamebirds for “put and take” shooting (Sokos et al., 2008), to conservation programs aiming to restore self‐sustaining populations. Efforts should be redirected toward habitat improvement through selected agricultural practices, to greatly increase refuge covers, insects, and seeds in the landscape, with hunting restrictions to ensuring that the bird populations are self‐sustaining, coupled with releases of wild‐caught or predator‐trained birds (Sokos et al., 2008). Creating synergy between all stakeholders, hunters, farmers, and scientists is, however, critical to achieve such integrated, local biodiversity management.

## CONFLICT OF INTEREST

None declared.

## AUTHOR CONTRIBUTIONS

VB set up the long‐term partridge and land‐cover monitoring; numerous fieldworkers supervised by VB collected the data. OP, VB, MS, and CH conceived the ideas and designed methodology; CH and OP analyzed the data; CH, OP, VB, and MS wrote the manuscript. All authors contributed to the drafts and gave final approval for publication.

## Data Availability

Partridge sightings and landscape metrics: OSF https://doi.org/10.17605/OSF.IO/QJ237.
