# Supplementary material for: Changes in habitat selection patterns of the gray partridge Perdix perdix in relation to agricultural landscape dynamics over the past two decades
Source: Ecol Evol. 2019 Apr 3;9(9):5236–47. doi: 10.1002/ece3.5114 (PMC6509401; doi:10.1002/ece3.5114)
Supplement: Supplementary file 1 [file ECE3-9-5236-s001.docx]

**Supporting information**

**Appendix A. Supplementary figures, tables and supplementary results from models calibrated on all sightings (opportunistic observations, point counts, point counts with call playback).**


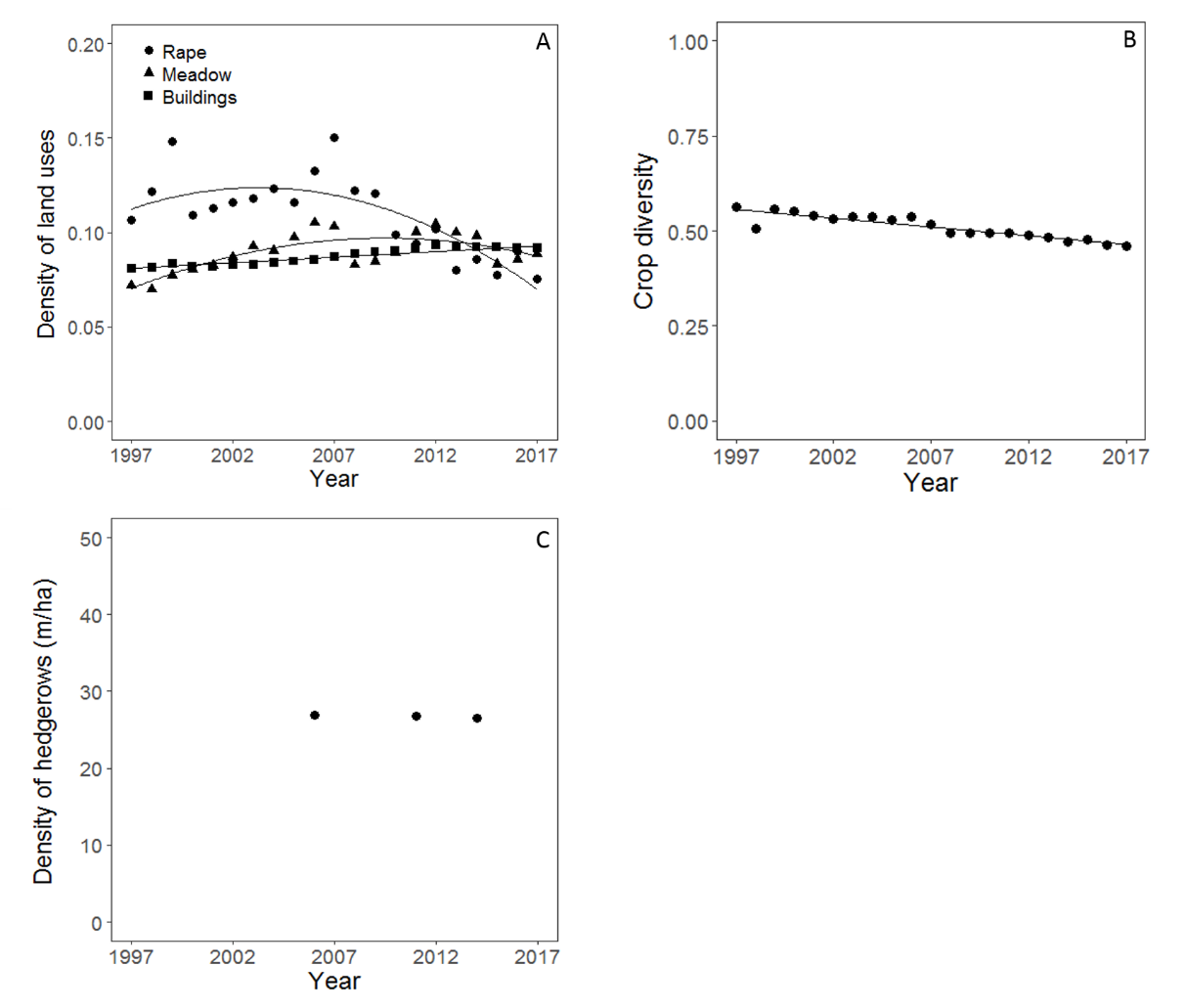


Figure S1. Dynamic of landscape features from 1997 to 2017. A: density of land covers; B: crop diversity; C: density of hedgerows (m/ha). Solid lines represent significant trends from GLS model with autoregressive moving average (ARMA) (see Table S1 for statistical details). For the density of hedgerows, values were only available for 2006, 2011 and 2014.


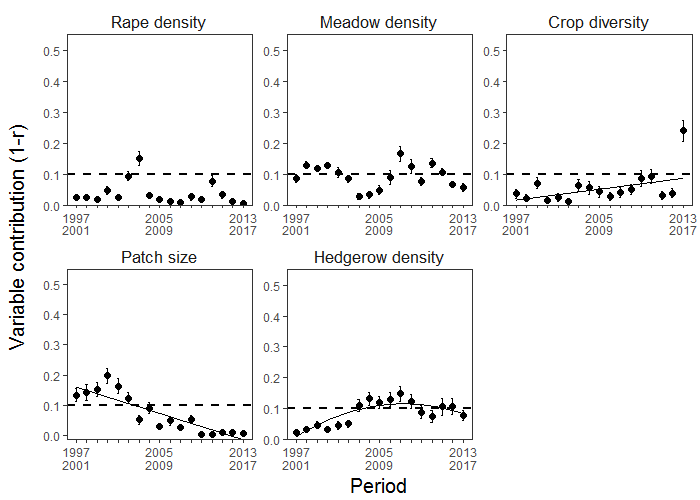


*Figure S2. Contributions (black dots) of landscape metrics in 5-year moving windows from 1997-2001 to 2013-2017). Solid lines correspond to significant trends from GLS model with autoregressive moving average (ARMA) (see Table S4 for statistical details). The dashed line represents the threshold used to distinguish the landscape metrics that contributed most to the model. Error bars show the standard deviations.*

*Table S1.* *Summary of the results of the generalized least squares models with Auto Regressive Moving Average (ARMA), showing the time dependence of the landscape metrics. Significant trends are represented by solid lines in Figures S1.*

| Landscape metric | Type of autocorrelation | Independent variable | F value | numDf | p value | Coef ± SE |
| --- | --- | --- | --- | --- | --- | --- |
| Cereal | ARMA(q=3) | (Intercept) |  |  |  | (0.3310 ± 0.0121) |
|  |  | Time | 1.864 | 1 | <0.001 | 0.0095 ± 0.0025 |
|  |  | Time² | 5.055 | 1 | 0.037 | -0.0002 ± 0.0001 |
|  |  |  |  |  |  |  |
| Rape | ARMA(q=2) | (Intercept) |  |  |  | (0.1086 ± 0.0135) |
|  |  | Time | 9.167 | 1 | 0.007 | 0.0042 ± 0.0028 |
|  |  | Time² | 5.255 | 1 | 0.034 | -0.0003 ± 0.0001 |
|  |  |  |  |  |  |  |
| Meadow | ARMA(q=1) | (Intercept) |  |  |  | (0.0659 ± 0.0062) |
|  |  | Time | 6.389 | 1 | 0.021 | 0.0046 ± 0.0013 |
|  |  | Time² | 9.086 | 1 | 0.008 | -0.0002 ± 0.0001 |
|  |  |  |  |  |  |  |
| Crop diversity | ARMA(q=3) | (Intercept) |  |  |  | (0.562 ± 0.007) |
|  |  | Time | 64.645 | 1 | <0.001 | -0.005 ± 0.001 |
|  |  | Time² | 2.222 | 1 | 0.153 |  |
|  |  |  |  |  |  |  |
| Patch size | ARMA(q=4) | (Intercept) |  |  |  | (4.637 ± 0.088) |
|  |  | Time | 136.948 | 1 | <0.001 | 0.079 ± 0.007 |
|  |  | Time² | 2.080 | 1 | 0.167 |  |
|  |  |  |  |  |  |  |
| Woodland | ARMA(q=1) | (Intercept) |  |  |  | (0.0314 ± 0.0003) |
|  |  | Time | 2.000 | 1 | 0.175 | -0.0003 ± 0.0001 |
|  |  | Time² | 22.700 | 1 | <0.001 | 0.00001 ± 0.0000 |
|  |  |  |  |  |  |  |
|  |  |  |  |  |  |  |
| Buildings | ARMA(q=4) | (Intercept) |  |  |  | (0.080 ± 0.001) |
|  |  | Time | 57.718 | 1 | <0.001 | 0.001 ± 0.000 |
|  |  | Time² | 0.910 | 1 | 0.353 |  |

Table S2. Metrics used in this study, how they were obtained, the expected relationship with the probability of occurrence of grey partridge, and the underlying mechanism accounting for this effect.

| Landscape metric | Calculation | Expected sign of the relationship | Underlying mechanism and reference |
| --- | --- | --- | --- |
| Cereal density | $\frac{Area of cereals}{pixel area}$ | + | Nesting site selection (Bro et al., 2013) |
| Rape density | $\frac{Area of rape}{pixel area}$ | + | Escape and food supply covers (Birkan et al., 1992) |
| Meadow density | $\frac{Area of meadows}{pixel area}$ | + | Nesting site selection (Bro et al., 2013) |
| Crop diversity | Shannon Index for each pixel (H= -∑p_i_*ln(p_i_) with p_i_ the proportion of crop i in the pixel) based on the six more represented categories of crops: cereals, sunflower, maize, rape, meadow, and other crops (Bertrand et al. 2016) | + | Nesting site selection (Reitz et al., 2002) |
| Patch size | Cultivated fields were aggregated in six categories of crops (cereals, sunflower, maize, rape, meadow, and other crops), and then divided by roads/tracks, and the average size of the resulting patches intersecting each pixel was calculated (Bertrand et al. 2016) | - | Nesting site selection (Kaiser, 1998) |
| Hedgerow density | $\frac{Length of hedgerows}{pixel area}$ | - | Open farmland species preferring fragmented rather than complete hedgerows (Blank et al., 1967) |
| Road density | $\frac{Length of roads}{pixel area}$ | + | Nesting site selection near linear features (Bro et al., 2013)  Seed accumulation in roadside habitats (von der Lippe et al., 2013) |
|  |  | - | High road density increases the predation risk by mustelids (Reitz & Mayot, 1999) |
| Distance to woodland | Distance from the centre of each pixel to the closest woodland | - | Avoidance of woodlands as predator reservoirs (foxes and mustelids; Dudzinski, 1992) |
| Distance to buildings | Distance from the centre of each pixel to the closest building | - | Avoidance of buildings as predator reservoirs (cats, dogs, mustelids) (Putaala, Turtola, & Hissa, 2001; Reitz et al., 2002) |
|  |  |  |  |

| year | TSS | Cereal density | Rape density | Meadow density | Crop diversity | Patch size | Hedgerow density | Distance to woodland | Road density | Distance to building |
| --- | --- | --- | --- | --- | --- | --- | --- | --- | --- | --- |
| 1997-2001 | **0.44** (± 0.05) | **0.21** (± 0.03) | 0.02 (± 0.01) | 0.09 (± 0.01) | 0.04 (± 0.01) | **0.13** (± 0.02) | 0.02 (± 0.01) | **0.21** (± 0.02) | 0.03 (± 0.01) | **0.14** (± 0.02) |
| 1998-2002 | **0.47** (± 0.04) | **0.26** (± 0.03) | 0.03 (± 0.01) | **0.13** (± 0.01) | 0.02 (± 0.01) | **0.14** (± 0.03) | 0.03 (± 0.01) | **0.16** (± 0.02) | 0.03 (± 0.01) | **0.22** (± 0.02) |
| 1999-2003 | **0.44** (± 0.05) | **0.18** (± 0.03) | 0.02 (± 0.01) | **0.12** (± 0.01) | 0.07 (± 0.02) | **0.15** (± 0.02) | 0.05 (± 0.01) | **0.15** (± 0.02) | 0.02 (± 0.01) | **0.27** (± 0.02) |
| 2000-2004 | **0.46** (± 0.05) | **0.24** (± 0.03) | 0.05 (± 0.01) | **0.13** (± 0.01) | 0.01 (± 0.01) | **0.20** (± 0.02) | 0.03 (± 0.01) | **0.12** (± 0.02) | 0.03 (± 0.01) | **0.23** (± 0.03) |
| 2001-2005 | **0.45** (± 0.05) | **0.11** (± 0.02) | 0.02 (± 0.01) | **0.10** (± 0.02) | 0.02 (± 0.01) | **0.16** (± 0.02) | 0.05 (± 0.01) | **0.16** (± 0.02) | 0.08 (± 0.02) | **0.20** (± 0.02) |
| 2002-2006 | **0.45** (± 0.05) | **0.26** (± 0.03) | 0.09 (± 0.02) | 0.09 (± 0.01) | 0.01 (± 0.01) | **0.12** (± 0.02) | 0.05 (± 0.01) | **0.15** (± 0.02) | 0.07 (± 0.02) | **0.12** (± 0.02) |
| 2003-2007 | **0.44** (± 0.05) | **0.13** (± 0.03) | **0.15** (± 0.02) | 0.03 (± 0.01) | 0.06 (± 0.02) | 0.05 (± 0.01) | **0.11** (± 0.02) | **0.19** (± 0.02) | 0.09 (± 0.02) | 0.09 (± 0.02) |
| 2004-2008 | **0.42** (± 0.05) | **0.11** (± 0.03) | 0.03 (± 0.01) | 0.03 (± 0.01) | 0.06 (± 0.02) | 0.09 (± 0.02) | **0.13** (± 0.02) | **0.17** (± 0.03) | **0.14** (± 0.02) | **0.12** (± 0.02) |
| 2005-2009 | 0.37 (± 0.05) | **0.20** (± 0.04) | 0.02 (± 0.01) | 0.05 (± 0.02) | 0.04 (± 0.02) | 0.03 (± 0.01) | **0.12** (± 0.02) | **0.17** (± 0.03) | **0.13** (± 0.03) | 0.07 (± 0.02) |
| 2006-2010 | 0.35 (± 0.05) | **0.14** (± 0.03) | 0.01 (± 0.01) | 0.09 (± 0.02) | 0.03 (± 0.01) | 0.05 (± 0.01) | **0.13** (± 0.02) | **0.15** (± 0.03) | **0.10** (± 0.02) | 0.07 (± 0.02) |
| 2007-2011 | 0.39 (± 0.05) | **0.20** (± 0.04) | 0.01 (± 0.01) | **0.17** (± 0.02) | 0.04 (± 0.02) | 0.03 (± 0.01) | **0.15** (± 0.02) | **0.10** (± 0.02) | **0.15** (± 0.03) | 0.06 (± 0.02) |
| 2008-2012 | **0.40** (± 0.05) | **0.17** (± 0.03) | 0.03 (± 0.01) | **0.12** (± 0.02) | 0.05 (± 0.02) | 0.00 (± 0.01) | **0.12** (± 0.02) | **0.11** (± 0.02) | **0.15** (± 0.02) | 0.07 (± 0.01) |
| 2009-2013 | 0.38 (± 0.05) | **0.24** (± 0.04) | 0.02 (± 0.01) | 0.08 (± 0.01) | 0.09 (± 0.02) | 0.00 (± 0.00) | 0.09 (± 0.02) | **0.13** (± 0.02) | **0.16** (± 0.03) | 0.08 (± 0.02) |
| 2010-2014 | **0.40** (± 0.06) | **0.26** (± 0.05) | 0.08 (± 0.02) | **0.14** (± 0.02) | 0.09 (± 0.02) | 0.01 (± 0.00) | 0.08 (± 0.02) | **0.10** (± 0.02) | **0.19** (± 0.03) | 0.04 (± 0.01) |
| 2011-2015 | **0.42** (± 0.06) | **0.31** (± 0.05) | 0.03 (± 0.01) | **0.11** (± 0.01) | 0.03 (± 0.01) | 0.01 (± 0.01) | **0.11** (± 0.03) | 0.07 (± 0.02) | **0.32** (± 0.04) | 0.07 (± 0.02) |
| 2012-2016 | **0.40** (± 0.05) | **0.38** (± 0.04) | 0.01 (± 0.01) | 0.07 (± 0.01) | 0.04 (± 0.01) | 0.01 (± 0.01) | **0.11** (± 0.03) | **0.14** (± 0.03) | **0.23** (± 0.03) | 0.03 (± 0.01) |
| 2013-2017 | **0.40** (± 0.05) | **0.31** (± 0.04) | 0.00 (± 0.00) | 0.06 (± 0.01) | **0.24** (± 0.03) | 0.01 (± 0.00) | 0.08 (± 0.02) | **0.15** (± 0.03) | **0.10** (± 0.03) | 0.04 (± 0.01) |
| *1997-2017* | ***0.43*** *(± 0.03)* | ***0.23*** *(±* *0.03)* | *-* | *0.04 (±* *0.01)* | *0.08* *(±* *0.02)* | *0.03* *(±* *0.01)* | *0.06 (±* *0.01)* | ***0.18*** *(±* *0.02)* | *0.08 (±* *0.02)* | ***0.12*** *(±* *0.02)* |

*Table S3. Mean TSS (± sd), and mean contribution of each metric (± sd) for each 5-year sliding window and for the whole period from 1997 to 2017. Values in bold represent TSS values above the threshold of 0.40 used to distinguish useful models and values above the threshold of 0.1 used to distinguish the metrics that contributed most to the model (Capinha & Anastácio, 2010; Leroy et al., 2013). Rape density was discarded from analyses of the whole period from 1997 to 2017 as it was correlated with cereal density.*

|  |  |  |  |  |  |  |  |  |  |  |
| --- | --- | --- | --- | --- | --- | --- | --- | --- | --- | --- |

*Table S4. Summary of the results of the generalized least squares models with Auto Regressive Moving Average (ARMA), showing the time dependence of the contribution of each landscape metric. Significant trends are represented by solid lines in Figs 4 & S2.*

| Landscape metric | Type of autocorrelation | Independent variable | F value | numDf | p value | Coef ±SE |
| --- | --- | --- | --- | --- | --- | --- |
| Cereal density | ARMA(q=1) | (Intercept) |  |  |  | (0.299 ± 0.018) |
|  |  | Time | 60.533 | 1 | <0.001 | -0.037 ± 0.005 |
|  |  | Time² | 96.173 | 1 | <0.001 | 0.002 ± 0.000 |
|  |  |  |  |  |  |  |
| Rape density | ARMA(q=1) | (Intercept) |  |  |  | (0.035 ± 0.014) |
|  |  | Time | 0.243 | 1 | 0.629 |  |
|  |  | Time² | 0.942 | 1 | 0.348 |  |
|  |  |  |  |  |  |  |
| Meadow density | ARMA(q=1) | (Intercept) |  |  |  | (0.090 ± 0.017) |
|  |  | Time | 0.080 | 1 | 0.782 |  |
|  |  | Time² | 0.051 | 1 | 0.825 |  |
|  |  |  |  |  |  |  |
| Crop diversity | ARMA(q=1) | (Intercept) |  |  |  | (0.011 ± 0.015) |
|  |  | Time | 9.194 | 1 | 0.008 | 0.004 ± 0.001 |
|  |  | Time² | 3.449 | 1 | 0.085 |  |
|  |  |  |  |  |  |  |
| Patch size | ARMA(q=2) | (Intercept) |  |  |  | (0.171 ± 0.028) |
|  |  | Time | 16.993 | 1 | <0.001 | -0.011 ± 0.003 |
|  |  | Time² | 0.068 | 1 | 0.799 |  |
|  |  |  |  |  |  |  |
| Hedgerow density | ARMA(q=1) | (Intercept) |  |  |  | (-0.011 ± 0.023) |
|  |  | Time | 9.611 | 1 | 0.008 | 0.023 ± 0.006 |
|  |  | Time² | 10.070 | 1 | 0.007 | -0.001 ± 0.000 |
|  |  |  |  |  |  |  |
| Distance to woodland | ARMA(q=1) | (Intercept) |  |  |  | (0.184± 0.020) |
|  |  | Time | 5.219 | 1 | 0.037 | -0.004 ± 0.002 |
|  |  | Time² | 0.488 | 1 | 0.496 |  |
|  |  |  |  |  |  |  |
| Road density | ARMA(q=1) | (Intercept) |  |  |  | (0.011 ± 0.035) |
|  |  | Time | 11.605 | 1 | 0.004 | 0.012 ± 0.003 |
|  |  | Time² | 0.535 | 1 | 0.477 |  |
|  |  |  |  |  |  |  |
| Distance to buildings | ARMA(q=2) | (Intercept) |  |  |  | (0.203 ± 0.035) |
|  |  | Time | 9.786 | 1 | 0.007 | -0.010 ± 0.003 |
|  |  | Time² | 0.244 | 1 | 0.629 |  |

Table S5. Raw numbers (and associated %) of partridge sightings according to the range of values of each landscape metric, for each of four contiguous windows (1997-2001, 2002-2006, 2007-2011 and 2012-2016).

| Landscape metric | Range of value | 1997 – 2001 | 2002 – 2006 | 2007 – 2011 | 2012 – 2016 |
| --- | --- | --- | --- | --- | --- |
| Cereal density | < 0.25 | 30 (13%) | 22 (12%) | 18 (8%) | 9 (7%) |
|  | 0.25 – 0.75 | 200 (87%) | 162 (88%) | 188 (89%) | 110 (90%) |
|  | > 0.75 | 1 (0%) | 0 (0%) | 6 (3%) | 2 (2%) |
| Distance to woodland (m) | < 250 | 34 (15%) | 30 (16%) | 36 (17%) | 27 (22%) |
|  | 250 – 1000 | 168 (73%) | 139 (76%) | 163 (77%) | 91 (75%) |
|  | > 1000 | 29 (12%) | 15 (8%) | 13 (6%) | 3 (2%) |
| Road densities (m/ha) | < 20 | 42 (18%) | 31 (17%) | 36 (17%) | 13 (11%) |
|  | 20 – 200 | 187 (81%) | 153 (83%) | 176 (83%) | 108 (89%) |
|  | > 200 | 2 (1%) | 0 (0%) | 0 (0%) | 0 (0%) |
| Distance to buildings (m) | < 160 | 29 (13%) | 37 (20%) | 34 (16%) | 26 (21%) |
|  | > 160 | 202 (87%) | 147 (80%) | 178 (84%) | 95 (79%) |

Table S6. Ranges and means of landscape metrics above the cutoff used to identify values of landscape metrics selected by grey partridge (see Methods for details) in four contiguous five-year periods.

| Period | 1997 – 2001 | 2002 – 2006 | 2007 – 2011 | 2012 – 2016 |
| --- | --- | --- | --- | --- |
| Cutoff | 0.564 | 0.515 | 0.483 | 0.496 |
| Range of selected cereal densities | 0.24-0.85 | 0.18-0.75 | 0.23-1.00 | 0.30-0.77 |
| Mean selected cereal density | 0.55 | 0.47 | 0.62 | 0.54 |
| Range of selected distances to woodland (m) | 340-1285 | 175-1350 | 170-1700 | 250-1060 |
| Mean selected distance to woodland (m) | 820 | 765 | 935 | 660 |
| Range of selected road densities (m/ha) | <270 | <200 | 20-170 | 35-165 |
| Mean selected road density (m/ha) | 137 | 99 | 96 | 99 |
| Range of selected distances to nearest building (m) | >160 | >0 | >0 | >0 |
| Mean selected distance to nearest building (m) | 1010 | 910 | 910 | 910 |

*Table S7. Mean size (min; max) and mean habitat suitability Index of suitable patches for grey partridge over time. The similarity between habitat suitability maps of 1997 – 2001 (taken as reference) and each subsequent five-year window was assessed as Schoener’s D. The threshold used to discriminate suitable from unsuitable patches is the cutoff of models calibrated for 1997-2001, i.e. 0.564 (see section Material and Methods).*

| Time period | Mean size (min; max) of suitable patches | Mean habitat suitability index of suitable patches | Schoener’s D |
| --- | --- | --- | --- |
| 1997-2001 | 26.40 ha (4; 1280) | 0.696 | – |
| 1998-2002 | 26.15 ha (4; 1416) | 0.697 | 0.945 |
| 1999-2003 | 25.98 ha (4; 1080) | 0.693 | 0.928 |
| 2000-2004 | 25.01 ha (4; 1120) | 0.695 | 0.906 |
| 2001-2005 | 23.73 ha (4; 1112) | 0.697 | 0.893 |
| 2002-2006 | 24.18 ha (4; 1212) | 0.697 | 0.872 |
| 2003-2007 | 24.12 ha (4; 1328) | 0.697 | 0.865 |
| 2004-2008 | 24.83 ha (4; 1848) | 0.694 | 0.853 |
| 2005-2009 | 23.76 ha (4; 1560) | 0.692 | 0.848 |
| 2006-2010 | 23.54 ha (4; 1408) | 0.692 | 0.840 |
| 2007-2011 | 22.04 ha (4; 968) | 0.689 | 0.835 |
| 2008-2012 | 21.05 ha (4; 916) | 0.688 | 0.825 |
| 2009-2013 | 21.66 ha (4; 936) | 0.687 | 0.821 |
| 2010-2014 | 21.05 ha (4; 1088) | 0.689 | 0.816 |
| 2011-2015 | 20.51 ha (4; 1152) | 0.685 | 0.814 |
| 2012-2016 | 20.63 ha (4; 1180) | 0.687 | 0.813 |
| 2013-2017 | 19.60 ha (4; 992) | 0.682 | 0.815 |

**Appendix B. Selection of uncorrelated explanatory variables**.

Explanatory variables, especially when numerous, are potentially highly intercorrelated (Barbet-Massin & Jetz, 2014). We used the method published by Leroy et al. (2013) to select uncorrelated variables (see Table S1 for details of the variables used): (1) identify groups of intercorrelated variables using a hierarchical ascendant classification on a distance metric calculated from the Pearson’s correlation coefficient; (2) within each group of intercorrelated variables, select the variable having the highest contribution to the prediction of occurrence.

As this selection procedure was performed on a dataset with pixels with sightings (presence) plus the pixels selected as pseudo-absences (Leroy et al., 2013), the results can be sensitive to the number of pseudo-absences generated. Barbet-Massin et al. (2012) recommend generating either 1 dataset with 10000 pseudo-absences or at least 10 datasets with 1000 pseudo-absences. The correlated groups were identified using both numbers of pseudo-absences.

1. Identification of groups of intercorrelated landscape metrics

From the presence and pseudo-absence pixels, we calculated the Pearson’s correlation coefficient (r) between each pair of variables. We then performed a hierarchical ascendant classification using a distance metric (1-r) to identify groups of intercorrelated variables. Leroy et al. (2013) used a correlation threshold of 0.7 (distance < 0.3) above which variables are considered as intercorrelated. In our analysis we will consider a more restrictive correlation threshold of 0.5 (distance < 0.5) as recommended by Barbet-Massin & Jetz (2014) to minimize correlation between predictors. This procedure was carried out for the whole 21-year period (1997-2017), and also for the 17 five-year windows (from 1997-2001 to 2013-2017), to ensure that variables were not intercorrelated over the study period. For either number of pseudo-absences, all variables were considered as uncorrelated for the 5-year moving windows, but cereal density and rape density were correlated for the whole 21-year period.

1. Selection of variables within the groups of intercorrelated variables

This second step was only carried out for the whole 21-year period as the variables were not correlated in the 5-year windows. To identify the highest contributive variable within each group of intercorrelated variables, models were calibrated with only the intercorrelated variables and their contributions were assessed using the *biomod2* *variables_importance* function (Leroy et al., 2013)*.* For a given variable, a standard prediction is made with a model calibrated with the intercorrelated variables, and then a series of predictions are made with randomized values of the given variable. The contribution of the variable to each prediction is 1 less the correlation with the standard prediction; the higher the correlation, the weaker the contribution or predictive power of the variable. Ten replications of the randomization procedure were used (Leroy et al., 2013). Finally, the variable with the highest contribution was selected.

Cereal density (contribution of 0.56 for 1000 pseudo-absences and 0.55 for 10000 pseudo-absences generated) was kept while rape density (contribution of 0.37 for 1000 pseudo-absences and 0.47 for 10000 pseudo-absences generated) was discarded. This choice is also consistent with the known biology of partridges which prefer cereal cover during the breeding period.

**Appendix C. Selection of the method for pseudo-absence generation, the number of pseudo-absences, and the modeling technique**

Modeling techniques of habitat suitability require presence and absence data. As our dataset did not contain true absences, pseudo-absences were generated. One recommendation is to generate either 10000 pseudo-absences in one dataset or at least 1000 pseudo-absences in 10 datasets by random sampling (Barbet-Massin et al., 2012). This method has been criticized for introducing bias (Phillips et al., 2009) so we also used the “disk” method in *biomod2* to generate pseudo-absences in areas which had been prospected and where no presence were recorded: within a radius of 1 km around pixels with presences (Phillips et al., 2009; Zaniewski, Lehmann, & Overton, 2002).

Then, to produce response curves, we had to select a modeling technique. The modeling technique must be selected according to the predictive performance, the ease of use, the comprehensibility, and the ease of interpretation of the outputs (Elith et al., 2005). In this step, seven modeling techniques commonly used in *biomod2* were tested: three used regressions, generalized linear model (GLM), generalized additive model (GAM), multiple adaptive regression splines (MARS); two used classifications: classification tree analysis (CTA), flexible discriminant analysis (FDA); and two used machine-learning: random forest (RF) and generalized boosting model (GBM) (Barbet-Massin et al., 2012; Leroy et al., 2013; Monnet et al., 2015). Models were calibrated with default options for all the uncorrelated explanatory variables. The true skill statistic (TSS) (Allouche et al., 2006) is commonly used for species distribution model evaluation.

TSS = specificity + sensitivity – 1, where the specificity is the proportion of absences well predicted by the model, and the sensitivity is the proportion of presences well predicted by the model (Allouche et al., 2006). The TSS / sensitivity pair can be used to select a model (Barbet-Massin et al., 2012). A modeling technique with a high TSS and a high sensitivity will be preferred to a modeling technique with a high TSS and a high specificity. The TSS and the sensitivity were assessed for each of the modeling techniques, for all combinations of the pseudo-absence generation method (“random”, “disk”) and the number of pseudo-absences generated per dataset (1000, 10000) (Table S8).

Firstly, for any period, either number of pseudo-absences, and any type of modeling technique, the TSS and sensitivity were higher when pseudo-absences were generated by random sampling (Table S8). This is consistent with the recommendation to prefer random sampling when true absences are unavailable (Barbet-Massin et al., 2012). Therefore, random sampling was selected.

Secondly, for any period, when pseudo-absences were randomly generated, the number of pseudo-absences per dataset had little effect on the TSS and sensitivity of the models, but 10 datasets of 1000 pseudo-absences seemed to give slightly better and more stable results over the study periods (Table S8). As generating of 1000 pseudo-absences in each dataset provides replication, and is faster, we used datasets with 1000 random pseudo-absences as is usual (Leroy et al., 2013; Monnet et al., 2015).

Thirdly, we selected a model using two criteria. The first was its suitability for drawing response curves: this requires a good predictive performance (Elith et al., 2005) and stability over the study period. Overall, all the modeling techniques had a similar predictive performance (Table S8). The regression techniques were more stable for both TSS and sensitivity and less influenced by the number of pseudo-absences per dataset. These results are consistent with Barbet-Massin et al. (2012) who found that GLMs and GAMs are not influenced as much as other modeling techniques by the number of pseudo-absences. These methods yielded the best sensitivity too.

The second criterion is that the technique should be easy to use and understand (Elith et al., 2005). As the shape of the response curves and the variation in model coefficients with time were the main outputs on interest, GLMs and GAMs are to be preferred as the predicted response to each variable does not depend on the other variables, unlike modeling technique with no inherent structure, or regression trees and MARS with interaction terms that may not be fitted to the entire range of each variable but on several segments of the variable (Elith et al. 2005). For these reasons, we selected GLM which was stable, performed as well as the others, had one of the best sensitivities, is easy to use and understand, is easy to parameterize, provides easy interpretable response curves (Elith et al. 2005) and is compatible with datasets with 1000 randomly generated pseudo-absences (Barbet-Massin et al., 2012). In order to obtain reliable confidence intervals, 100 datasets were used in the final modeling process.

*Table S8. Performance of the various modeling techniques (GLM, GAM, MARS, CTA, FDA, RF, GBM) and average performance over these techniques take together (“mean”) measured as the TSS (true skill statistic) and the sensitivity as a function of the method used to generate pseudo-absences, the number of pseudo-absences, and the period*

|  |  | **Random** | | | | | | | | | | **Disk** | | | | | | | | | |
| --- | --- | --- | --- | --- | --- | --- | --- | --- | --- | --- | --- | --- | --- | --- | --- | --- | --- | --- | --- | --- | --- |
|  |  | 1997-2001 | | 2002-2006 | | 2007-2011 | | 2012-2016 | | 1997-2017 | | 1997-2001 | | 2002-2006 | | 2007-2011 | | 2012-2016 | | 1997-2017 | |
|  |  | TSS | Sens. | TSS | Sens. | TSS | Sens. | TSS | Sens. | TSS | Sens. | TSS | Sens. | TSS | Sens. | TSS | Sens. | TSS | Sens. | TSS | Sens. |
| **10*1,000 PA** | GLM  GAM  MARS  CTA  FDA  RF  GBM  ***mean*** | 0.43  0.44  0.45  0.35  0.44  0.49  0.48  ***0.44*** | 70.36  73.61  71.97  69.72  70.98  73.25  66.10  ***70.98*** | 0.44  0.43  0.42  0.38  0.40  0.46  0.49  ***0.43*** | 76.92  75.77  75.77  66.42  75.72  69.95  77.01  ***73.94*** | 0.40  0.36  0.37  0.33  0.39  0.42  0.43  ***0.39*** | 74.29  74.81  72.34  67.75  71.30  70.04  76.62  ***72.45*** | 0.42  0.40  0.35  0.33  0.37  0.42  0.46  ***0.39*** | 77.20  78.94  77.88  64.24  74.85  65.45  74.62  ***73.31*** | 0.43  0.44  0.44  0.41  0.43  0.52  0.47  ***0.45*** | 74.98  75.62  76.24  73.81  76.46  72.69  77.68  ***75.35*** | 0.38  0.36  0.38  0.30  0.37  0.42  0.42  ***0.38*** | 66.91  64.02  68.92  63.61  66.63  65.66  70.32  ***66.58*** | 0.37  0.35  0.33  0.31  0.33  0.39  0.39  ***0.35*** | 66.72  70.95  67.71  62.44  65.92  60.55  67.96  ***66.03*** | 0.33  0.31  0.26  0.27  0.32  0.35  0.36  ***0.31*** | 72.99  68.92  62.42  64.59  69.00  56.93  67.97  ***66.12*** | 0.35  0.35  0.29  0.28  0.29  0.38  0.40  ***0.34*** | 77.73  75.00  70.98  61.59  68.86  60.23  70.61  ***69.29*** | 0.38  0.38  0.38  0.36  0.39  0.49  0.42  ***0.40*** | 71.02  72.59  73.29  71.96  70.90  68.12  74.36  ***71.75*** |
| **1*10,000 PA** | GLM  GAM  MARS  CTA  FDA  RF  GBM  ***mean*** | 0.44  0.45  0.45  0.32  0.40  0.40  0.49  ***0.42*** | 71.89  77.91  79.91  59.04  71.08  73.09  75.10  ***70.97*** | 0.44  0.43  0.41  0.41  0.38  0.430.47  ***0.42*** | 73.13  77.11  78.61  68.66  73.63  60.20  76.12  ***72.49*** | 0.36  0.36  0.34  0.36  0.38  0.35  0.41  ***0.36*** | 65.80  75.76  59.74  65.80  67.10  65.37  69.70  ***67.04*** | 0.37  0.39  0.40  0.34  0.30  0.25  0.45  ***0.36*** | 76.52  75.00  73.49  61.36  69.70  56.82  75.00  ***69.70*** | 0.42  0.45  0.44  0.42  0.42  0.50  0.48  ***0.45*** | 77.48  79.50  76.35  63.96  73.42  61.49  81.08  ***73.33*** | 0.39  0.37  0.37  0.29  0.38  0.39  0.43  ***0.37*** | 70.28  69.48  65.46  50.60  71.89  68.27  70.28  ***66.61*** | 0.38  0.30  0.32  0.36  0.34  0.33  0.38  ***0.34*** | 67.16  76.12  69.65  60.70  70.65  45.77  81.59  ***67.38*** | 0.35  0.27  0.33  0.30  0.29  0.32  0.36  ***0.32*** | 65.80  59.31  63.20  75.32  59.31  51.95  56.71  ***61.66*** | 0.31  0.33  0.34  0.25  0.30  0.41  0.39  ***0.33*** | 81.82  78.03  77.27  59.85  69.70  59.09  65.91  ***70.24*** | 0.39  0.38  0.39  0.41  0.37  0.49  0.43  ***0.41*** | 75.45  73.87  70.95  69.48  66.67  64.08  75.90  ***70.91*** |
